# Structure design and photocatalytic properties of one-dimensional SnO_2_-TiO_2_ composites

**DOI:** 10.1186/s11671-015-0901-8

**Published:** 2015-04-28

**Authors:** Yuan Chen, Bitao Liu, Junfang Chen, Liangliang Tian, Lei Huang, Mingjing Tu, Shuai Tan

**Affiliations:** School of Material Science and Engineering, Chongqing University of Technology, 69 Hongguang Street, Banan, Chongqing, 400054 China; Research Institute for New Materials Technology, Chongqing University of Arts and Sciences, 319 Honghe Street, Yongchuan, Chongqing, 402160 China; Carbon Neutral Energy Solutions Lab, Georgia Institute of Technology, 495 Tech Way NW, Atlanta, GA 30318 USA

**Keywords:** Structural, Nanocomposite, Porous materials

## Abstract

One-dimensional SnO_2_-TiO_2_ composites were prepared via emulsion electrospinning process. The obtained samples were characterized by a series of devices. The results showed that the porous core-shell SnO_2_-TiO_2_ photocatalyst exhibited enhanced photocatalytic activity on the degradation of methyl orange (MO). It should be ascribed to the novel structure, which could separate the electrons and holes effectively.

## Background

Nanophotocatalysis has been widely used as a useful technique in environmental cleaning [1]. The morphology of the nanosized materials would be the key factor for their photocatalytic activity. It is reported that one-dimensional nanomaterials have drawn particular attention because they can provide very high surface area and enhanced electron diffusion [2,3]. As well known, electrospinning was a simple and straightforward method to produce fiber structures with one-dimensional material, and it has attracted extensive attention in the past two decades [4]. As an improved special technology, emulsion electrospinning was proposed to be an efficient way to prepare heterostructure materials [5,6].

**Figure 1:**
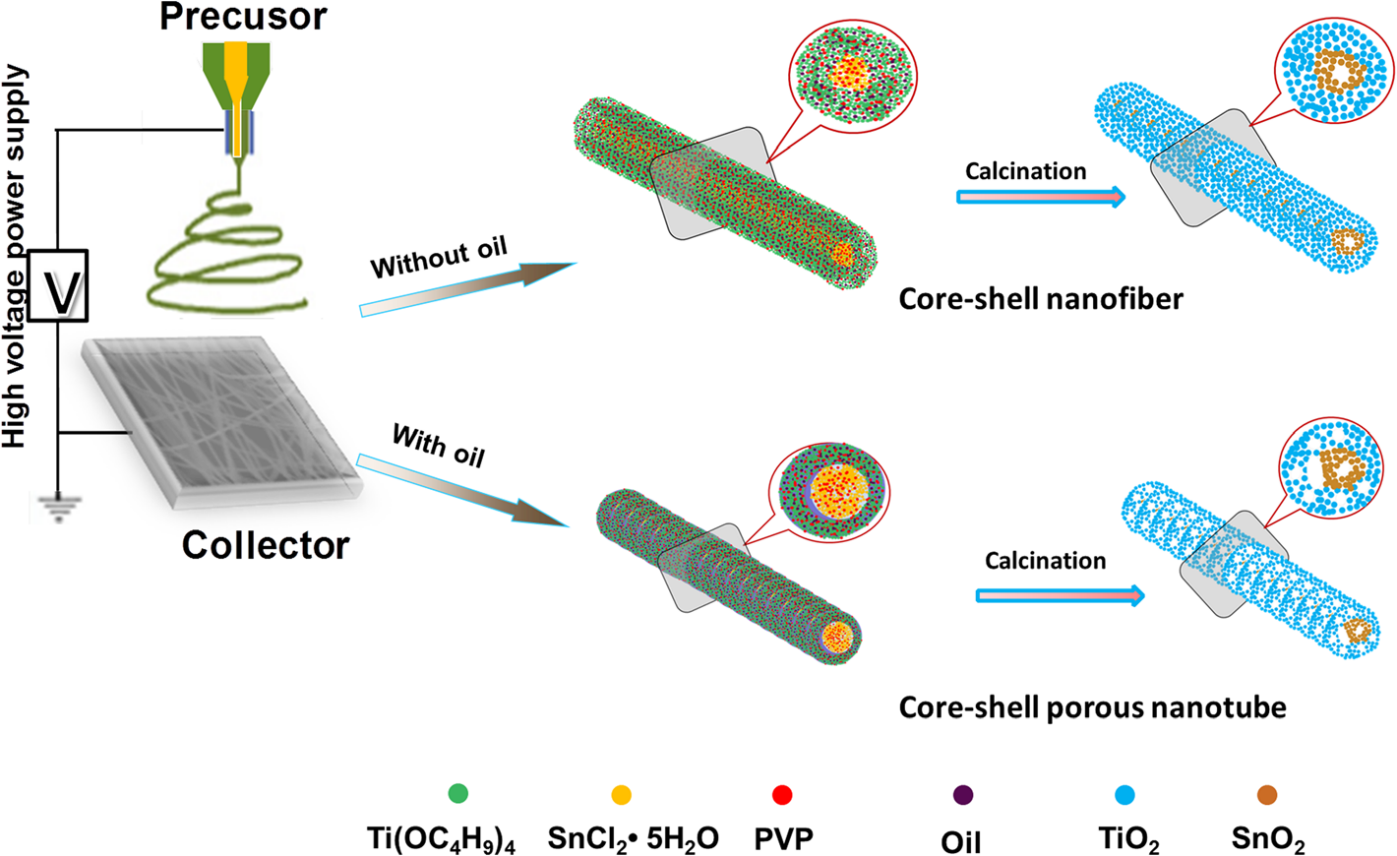
Illustration of the preparation of SnO_2_-TiO_2_ core-shell structure nanocomposite processes.

Herein, we try to design the SnO_2_-TiO_2_ heterostructure with different structures by an emulsion electrospinning process, and we also want to investigate the photocatalytic properties with different structures. The formation mechanisms of the core-shell structure composite are illustrated in Figure 1. As shown, once the fibers were spun out from the coaxial nozzle, the core-shell structure was directly formed. The oil would be the key factor to obtain a nanofiber (NF) or porous nanotube (NT) structure. These two structures were also different from the directly mixed nanofibers [7]. What is the difference in their photocatalytic properties? Thus, it would be interesting to compare these different-structured SnO_2_-TiO_2_ composites.

## Methods

### Reagents

All chemicals were of analytical grade and were used without further purification. Polyvinylpyrrolidone (PVP, 1,300,000), tetrabutyl titanate (C_16_H_36_O_4_Ti, 99.8%), stannous chloride (SnCl_2_ · 2H_2_O, reagent grade, ≥98.5%), N,N-dimethylformamide (DMF, ≥98%), acetylacetone (C_5_H_8_O_2_, ≥99%), ethanol (reagent grade, 99.5%), and mineral oil (SF 15 W-45, Sinopec, Beijing, China) were used. Distilled (DI) water was used as the solvent in this study.

### Synthesis of different-structured SnO_2_-TiO_2_ composites

At first, 6.03 g tetrabutyl titanate, 2.4 g DMF, 4 g alcohol, 0.8 g PVP, and 3 g mineral oil were added in turn and stirred for 4 h; 0.8 g SnCl_2_ · 2H_2_O (keep the mole ratio of Sn:Ti = 1:5), 4.4 g DMF, 4.4 g ethyl alcohol, 0.8 g PVP, and 0.3 g acetylacetone were added in turn in another beaker and stirred for 4 h. The electrospinning process was performed as reported [6]. The obtained fibers were maintained at 600°C for 2 h at a heat rate of 5°C/min. Additionally, the first solution was not added with any mineral oil; a couple of solutions were also fabricated by a directly mixed process.

### Instruments

The morphologies of different samples were observed by field emission scanning electron microscope (FESEM; S-4800, Hitachi, Chiyoda, Tokyo, Japan) and transmission electron microscopy (TEM). The crystal phase of the obtained materials was determined by powder X-ray diffraction (XRD; Rigaku D/MAX-2400 X-ray diffractometer (Rigaku, Tokyo, Japan) with Ni-filtered Cu Kα radiation). Infrared spectra were recorded using a Bruker VERTEX 70 FTIR spectrometer (Bruker Corporation, Billerica, MA, USA). The thermogravimetric (TG) analysis and differential thermal analysis (DTA) were performed with a PerkinElmer Diamond TG/DTA apparatus (PerkinElmer Inc., Waltham, MA, USA). TG and DTA were carried out simultaneously at a heating rate of 10°C/min in a flowing nitrogen atmosphere. UV-visible (UV-vis) diffuse reflectance spectra were recorded on a Shimadzu UV-3900 spectrophotometer (Shimadzu Co. Ltd., Beijing, China) with an integrating sphere, and BaSO_4_ was used as the reference. The photoelectric performance was measured using an electrochemical system (CHI-660B, Chenhua Instruments, Shanghai, China). A standard three-electrode cell with a working electrode (as-prepared photocatalyst), a platinum wire counter electrode, and a standard calomel electrode (SCE) as the reference electrode were used in photoelectric studies. In addition, 0.1 M Na_2_SO_4_ was used as the electrolyte solution. The potentials are given with reference to the SCE. The photoresponse of the photocatalysts in the presence and absence of UV light was measured at 0.0 V. Electrochemical impedance spectra (EIS) were recorded in the open-circuit potential mode.

### Catalyst activity measurement

The liquid-phase photodegradation of methyl orange (MO) was carried out in a quartz tube under the irradiation of UV light. In a typical process for degradation of MO under the UV irradiation, 200 mg of catalyst was suspended in 500 mL of 20 ppm dye solution. Before irradiation, the suspensions were stirred in the dark for 30 min to ensure the establishment of adsorption-desorption equilibrium. The quartz tube was exposed to the UV irradiation produced by a 500-W Xe arc lamp equipped with a band-pass light filter (400 nm). The solution was analyzed on a Varian UV-vis spectrophotometer (Cary-50, Varian Co., Palo Alto, CA, USA). The percentage of degradation is reported as *C*/*C*_0_. Here, *C* is the absorption of dye solution at each irradiated time interval of the main peak of the adsorption spectrum, while *C*_0_ is the absorption of the initial concentration when the adsorption-desorption equilibrium is reached.

## Results and discussion

**Figure 2:**
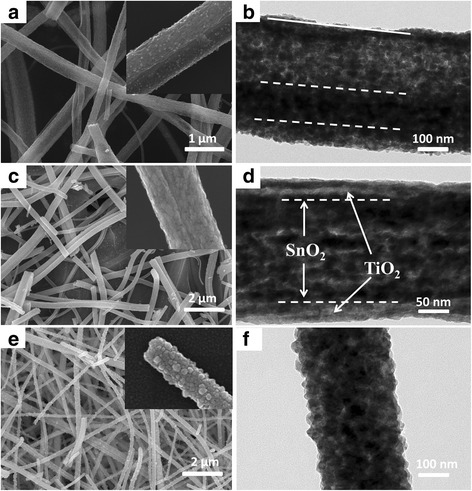
SEM and TEM images of different-structured SnO_2_-TiO_2_ composites. SEM image of **(a)** core-shell porous nanotube, **(c)** core-shell nanofibers, and **(e)** mixed nanofibers. TEM images of **(b)** core-shell porous nanotube, **(d)** core-shell nanofibers, and **(f)** mixed nanofibers.

The SEM and TEM images of the as-prepared samples are listed in Figure 2. As shown, the three morphologies were successfully obtained by the electrospinning process, and all the samples present a uniform one-dimensional structure with diameter at 400 ~ 600 nm. The tubular structure can be clearly seen in the insert of Figure 2a; obvious holes and phase interface can be found in Figure 2b. Figure 2c,d shows the core-shell structure nanofibers; the boundaries (marked in the line) between two phases of SnO_2_ and TiO_2_ could be directly observed. For mixed nanofibers (Mix-NF) sample (Figure 2f), no characteristic boundaries can be found, and the surface of the nanofiber was covered by many large grains, which should be the different growth processes of the two oxides during the annealing process.

**Figure 3:**
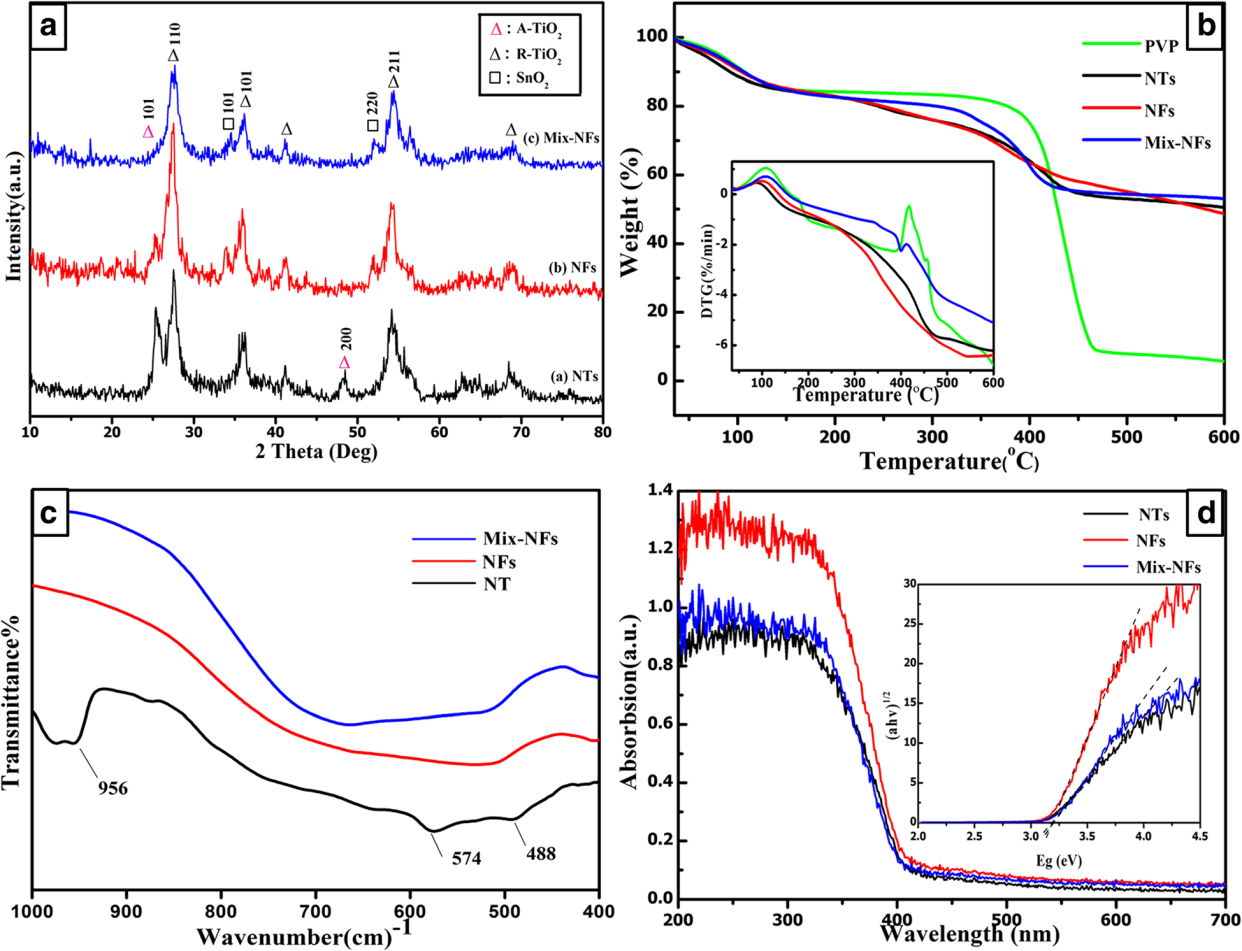
XRD, TG-DTG, FTIR, and UV-vis of different-structured SnO_2_-TiO_2_ composites. **(a)** XRD patterns, **(b)** TG-DTG curves, **(c)** FTIR spectra, and **(d)** UV-vis diffuse reflectance spectra.

Figure 3a shows the XRD patterns of series SnO_2_-TiO_2_ composites. All the peaks can be identified to TiO_2_ (anatase: JCPDS #71-1166, rutile: JCPDS #73-1232) and SnO_2_ (JCPDS #77-0451) phases. The peaks at 25.3° and 48.1° for the anatase phase TiO_2_ decrease significantly with the change of the structure from NTs to Mix-NFs. It means that the mineral oil would restrict the transformation of anatase to rutile phase [8]. Additionally, the peaks at 33.9° and 51.8° ascribed to SnO_2_ will increase with the structure change of the structure from NTs to Mix-NFs, indicating that the mineral oil may restrict the movement of Sn^4+^ to the surface.

Figure 3b shows the TG-DTG curves of the as-spun composites. A small weight loss (15%) at low temperature is observed due to the release of DMF and alcohol. A large weight loss (40% ~ 80%) at the range of 300°C to 470°C can be attributed to the decomposition of organic species such as PVP. Above 470°C, there was little weight loss, indicating the decomposition of the last organic species and the transformation of anatase TiO_2_ to rutile phase [9]. The DTG curve in the insert corresponds to the TG results.

The FTIR spectra of the different structures of SnO_2_-TiO_2_ are presented in Figure 3c. As shown, a broad band at 700 and 400 cm^−1^ was assigned to Sn-O and Sn-O-Sn stretching vibrations, respectively [10]. Obviously, the width of Sn-O-Sn band changed with different structures. Such results may be attributed to the improvement in crystallinity, resulting from the removal of residual organic impurities [11]. It also can be seen that there was a band at 1,000 to 940 cm^−1^ in the nanotubes, and this band should be assigned to Ti-O-Ti stretching vibrations from anatase phase. However, this band did not exist in the other two nanofibers, which was consistent to the XRD results.

The UV-visible diffuse reflectance spectra at the wavelength range of 200 to 700 nm for the SnO_2_-TiO_2_ products are shown in Figure 3d. The absorption edges around 411 nm for these three different-structured samples could be observed, and these did not shift with the structure change, indicating that the different structures would not affect the band gap variation under the same Sn/Ti ratio.

**Figure 4:**
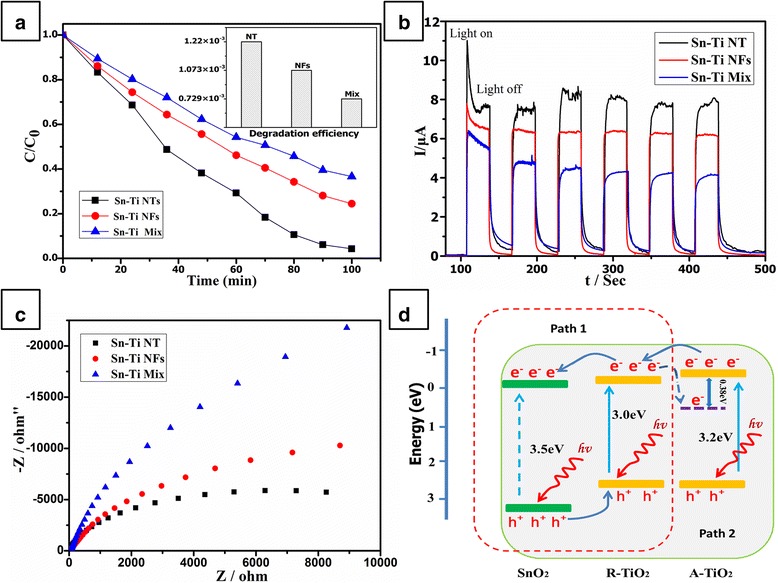
Photoelectrochemical properties and reaction mechanism of different-structured SnO_2_-TiO_2_ composites. **(a)** The liquid-phase photodegradation of MO under UV light irradiation of different samples; the insert shows the degradation efficiency of the unit area. **(b)** Transient photocurrent responses of different samples under Xe lamp irradiation. **(c)** Electrochemical impedance spectroscopy (EIS) Nyquist plot of NT, NF, and Mix samples, respectively. **(d)** A proposed schematic illustration showing the reaction mechanism for photocatalytic degradation of organic pollutants under UV light irradiation.

The photocatalytic activities of SnO_2_-TiO_2_ composites are shown in Figure 4a. Distinctly, the SnO_2_-TiO_2_ NT sample exhibited a much higher photocatalytic activity than the other two, which degraded 91% MO in 100 min while the other two samples were only 75% and 63%. The degradation efficiency was 0.0455, 0.0375, and 0.0315 mg/min, respectively. Actually, since the surface area of these three samples was 37.2554, 34.9239, and 43.1997 m^2^/g, the degradation efficiency of the unit area can be concluded as 1.22 × 10^−3^, 1.073 × 10^−3^, and 0.729 × 10^−3^ mg/min/m^2^, respectively (insert in Figure 4a). Obviously, the NT showed more effective photocatalytic activity of the unit area than the NF and Mix samples.

For a further investigation, some characteristic methods were used. It is well known that one-dimensional structure would form plenty of space-charge regions, which would provide an additional energetic barrier to recombination [12]. In addition, the charge transfer rate in TiO_2_ is about 0.1 to 1 cm^2^ V^−1^ s^−1^, which is much lower than that in SnO_2_ (100 to 200 cm^2^ V^−1^ s^−1^) [13]. Thus, due to the core-shell structure, the excited electrons would efficiently separate with the inner SnO_2_ phase and not recombine with the TiO_2_ phase. This was confirmed by the photocurrent response of serious samples as shown in Figure 4b. As well known, the photocurrent originated from the diffusion of the photogenerated electrons to the back contact. It clearly showed that the photocurrent response of these three samples was NT > NFs > Mix, indicating a more efficient separation and longer lifetimes of electron-hole pairs in Sn-Ti NT than NF and Mix samples. Furthermore, the EIS result also proves this efficient electron-hole separation process. It can be seen that the impedance arc radius of Sn-Ti nanotube is smaller than that of the NF and Mix samples under Xe lamp irradiation, which demonstrated an efficient electron-hole pair separation in Sn-Ti nanotube.

Thus, a proposed mechanism of the SnO_2_-TiO_2_ composite is shown schematically in Figure 4d. The band gap of anatase TiO_2_ and rutile SnO_2_ is 3.2 and 3.5 eV, respectively. The CB potential (*E*_CB_) of anatase TiO_2_ is −0.25 eV and rutile is −0.05 eV [14], while the CB potential of SnO_2_ is −0.01 eV. Due to the potential difference and different structures, we proposed two possible ways of electron transfer in these SnO_2_-TiO_2_ composites. For the mixed nanofibers, only SnO_2_ and rutile-phase TiO_2_ exist; thus, the electron transfer from the conduction band of rutile-phase TiO_2_ to SnO_2_ is shown as path 1 in Figure 4d. For the nanotubes, there should be mixed phase of TiO_2_ and SnO_2_ (as shown in Figure 3a); thus, the electrons would firstly transfer from rutile TiO_2_ to anatase TiO_2_. Subsequently, these electrons would transfer to anatase SnO_2_, and this multistep transfer should be more efficient than path 2. As depicted in our previous work, rutile crystallites in the mixed-phase TiO_2_ nanotubes would increase electron transfer from rutile to lower energy anatase lattice trapping sites, leading to a more stable charge separation [15]. This means that there would be more efficient charge separation in nanotubes than the mixed nanofibers, which well agrees with the transient photocurrent response and EIS results before. Therefore, the sample with few contents of anatase TiO_2_-enhanced photocatalytic activity of core-shell nanofibers can be understood.

## Conclusions

In summary, different structures of SnO_2_-TiO_2_ composite material were successfully fabricated via emulsion electrospinning technology. The results showed that this core-shell porous nanotube structure would enhance the photocatalytic degradation rate of MO, which can degrade 91% MO in 100 min higher than 75% and 63% for the other two samples. The photocurrent response and EIS results demonstrated that a much more efficient separation and longer lifetimes of electron-hole pairs existed in Sn-Ti nanotube, which should be responsible for the increased photocatalytic activities. This method could be extended to fabricate other composite materials.
